# ICU Physicians' Perception of Patients' Tolerance Levels in Light Sedation Impacts Sedation Practice for Mechanically Ventilated Patients

**DOI:** 10.3389/fmed.2019.00226

**Published:** 2019-10-18

**Authors:** Yichun Gong, Huilong Yang, Junqing Xie, Jingtao Liu, Jianxin Zhou, Penglin Ma

**Affiliations:** ^1^Medical School of Chinese PLA, Beijing, China; ^2^SICU, The Eighth Medical Center of PLA General Hospital, Beijing, China; ^3^ICU, Hebei Yanda Hospital, Langfang, China; ^4^Feng Tai District Center for Disease Control and Prevention, Beijing, China; ^5^NICU, Beijing Tiantan Hospital, Capital Medical University, Beijing, China

**Keywords:** physician's perception, light sedation, stimulus intensity, sedation practice, mechanical ventilation

## Abstract

**Purpose:** To investigate physicians' perception of patients' tolerance levels regarding sedation, which could affect sedation practice for mechanically ventilated (MV) patients.

**Methods:** This is a questionnaire survey combined with a 24 h cross-sectional study. The physician's propensity score for light sedation (PS-_LS_) was estimated by his/her response to the given answers for each item of the questionnaire, which tested the levels of interviewee's desire to manage MV patient with light sedation. Thereby, the mean physicians' PS-_LS_ of each participating ICU (ICU-_mean_PS-_LS_) was calculated. The practical measurements of all variables listed on the questionnaire were used to semi-quantitatively assess stimulus intensity of what the recruited patients suffered (i.e., semi-quantitative stimulus intensity, SSI). Sedation depth was assessed by Richmond Agitation Sedation Scale (RASS).

**Results:** 555 of 558 (99.5%) physicians from 102 ICUs were concerned with patients' tolerance levels regarding sedation while titrating sedation depth. The physician's PS-_LS_ was non-normally distributed with median (IQR) of 3 (0–5). ICU-_mean_PS-_LS_ was calculated in 92 out of 102 ICUs participating in the cross-sectional study, which was ranged from −5 to 7 with a median (IQR) of 2.37 (0.16–4.33). A significant increasing trend in prevalence of light sedation was observed over increasing ICU-_mean_PS-_LS_ quartiles (from Q1 to Q4, χ^2^-test for trend, *p* = 0.002). Moreover, odds ratio for probability of light sedation remained significant in MV patients from Q4 ICUs vs. Q1 ICUs, adjusted by APACHE II score (OR, 2.332; 95% CI: 1.463–3.717; p < 0.001) or SSI score (OR, 2.445; 95% CI: 1.468–4.074; *p* = 0.001). Notably, adjusted OR for mortality was significant in deeply sedated MV patients (OR, 2.034; 95% CI: 1.435–2.884; *p* < 0.001).

**Conclusions:** ICU physician's individualized perception for patients' tolerance levels regarding sedation, in light sedation affected sedation practice for MV patients.

## Introduction

While deep sedation has been associated with morbidity and mortality in mechanically ventilated (MV) patients (1–3), increasing data suggest that targeting lighter levels of sedation (RASS −2 ~ +1, Richmond Agitation Sedation Scale) could be beneficial to reduce ICU stay and ventilator days (4–6). The strategy of maintaining light rather than deep sedation was therefore recommended as a standard care for critically ill adult patients worldwide (7–9). However, recently published studies demonstrated that up to 65% MV patients continued to score deep sedation (RASS ≤ −3) (3, 10–12), which suggested that the recommendation for promoting lighter sedation is far from being implemented well.

Low adherence to lightening sedation strategy remained under interpreted, although frequent deep sedation was previously linked to inadequate assessments, lack of multidisciplinary cooperation, and even misperception as well (13–16). Interestingly, Rose et al. recently reported that most ICU nurses and physicians were concerned about agitation and agitated events, which affected their willingness to decrease sedation significantly (17). In fact, agitation was common in MV patients (18, 19). Moreover, agitation or agitated adverse events were observed more frequently in the arm of patients who were sedated at the lighter target than the usual care in several published randomized control trials (RCTs) (4–6, 20). Meanwhile, evidence regarding ICU physicians' concerns about patients' tolerance levels in light sedation remain limited. Notably, it was under investigated whether ICU physicians' concerns affected their behaviors in sedation titration. Therefore, a questionnaire survey combined with a cross-sectional study were conducted, hypothesizing that ICU physician's perception for patient's tolerance levels in light sedation was individualized, which impacted decision-making on implementation of minimizing sedation strategy for MV patients.

## Methods

A total of 102 Chinese ICUs, where members of the standing committees of Chinese Association of Critical Care Physicians or Chinese Society of Critical Care Medicine worked, were involved in this study. A questionnaire combined with a 24 h cross-sectional study was simultaneously conducted in each participating ICU on May 11th, 2016, approved by the Ethics Committees of each hospital. Owing to the absence of additional interventions, obtaining informed written consent for the individual patients was waived. The study protocol was registered on the website of www.chictr.org.cn (registration number: ChiCTR-EOC-16008444).

### Questionnaire Survey

A questionnaire named “Concerns of ICU physicians for lightening the sedation depth in MV patients” (Cronbach's Alpha = 0.737, Table 1) was developed by a modified Delphi method. The major components of the questionnaire were based on results of a three-round Delphi processing in a panel of 15 experts and testing in 63 doctors. Consequently, 10 stimuli-derived events (with agreement rate > 80%, Table S1) were selected, comprising two domains, ventilator setting and vital organ dysfunction. The ventilator settings included four stimuli-derived events, the ventilation mode (Mode), positive end-expiratory pressure (PEEP), plateau pressure (Pplat), and fraction of inspiration O_2_ (FiO_2_) as well. In the vital organ dysfunction domain, we examined the PaO_2_/FiO_2_ (P/F), respiratory rate (RR), minute ventilation (Min-V), plasma lactate level (Lac), Glasgow Coma Scale (GCS), and dosage of norepinephrine (NE) or equal-effect dose of other vasopressors. We allowed three response options (A, B, C), leading to the categorization of the response from high to low. An additional answer D was used to define an event that was not considered important in the decision-making for sedation depth. For conducting this questionnaire, up to six on-duty physicians (no more than two physicians with title of either senior, attending or resident, respectively) from each of participating ICUs were interviewed face-to-face by a well-trained clinical research coordinator on May 11th, 2016. All questionnaires were collected directly after interviewing and incomplete questionnaires were excluded from this study.

**Table 1:** Questionnaire items to address the ICU physicians concerns regarding lightening the sedation depth for MV patients.

**1. Ventilator settings domain**				
General question:	Did ventilator settings impact your option on titrating the depth of sedation for ventilated patients? Answers: A (Yes) □; B (Never)?			
Specific items: you wouldn't prefer to initiate light sedation if				
Mode	A. CMV □;	B. SIMV □;	C. PSV □;	D. Never mind □
PEEP (cmH_2_O)	A. ≥ 15 □;	B. ≥ 10 □;	C. ≥ 5 □;	D. Never mind □
Pplat (cmH_2_O)	A. ≥ 35 □;	B. ≥ 30 □;	C. ≥ 25 □;	D. Never mind □
FiO_2_ (%)	A. ≥ 60 □;	B. ≥ 50 □;	C. ≥ 40 □;	D. Never mind □
**2. Organ dysfunction domain**				
General question:	Did severity of organ dysfunction impact your option on titrating the depth of sedation for ventilated patients? Answers: A (Yes) □; B (Never) □			
Specific items: you wouldn't prefer to initiate light sedation if				
PO_2_/FiO_2_	A. ≤ 100 □;	B. ≤ 200 □;	C. ≤ 300 □;	D. Never mind □
RR (F/min)	A. ≥ 35 □;	B. ≥ 30 □;	C. ≥ 25 □;	D. Never mind □
Min-V (L/min)	A. ≥ 30 □;	B. ≥ 20 □;	C. ≥ 15 □;	D. Never mind □
NE (μg/kg/min)	A. ≥ 1.0 □;	B. ≥ 0.5 □;	C. ≥ 0.1 □;	D. Never mind □
Lac (mmol/l)	A. ≥ 6.0 □;	B. ≥ 4.0 □;	C. ≥ 2.0 □;	D. Never mind □
GCS	A. ≤ 8 □;	B. ≤ 10 □;	C. ≤ 12 □;	D. Never mind □

*The components of this questionnaire were classified into two domains, ventilator setting and organ dysfunction. A general question and four or six specific items were asked in each domain. If you choose answer “A” in the general question, please complete the follow-up questions for specific items. There were three given options for different levels of each item (answer A, B, and C), on which you wouldn't prefer to initiate light sedation. If you don't consider this item important for your decision, please select the answer “D.” Mode, ventilation mode; PEEP, positive end-expiratory pressure; Pplat, plateau pressure; FiO_2_, fraction of inspiration O_2_; RR, respiratory rate; Min-V, minute ventilation; NE, dosage of norepinephrine or equal-effect dose of other vasopressors; Lac, plasma lactate; GCS, Glasgow Coma Scale*.

Specifically, answer “A” was defined as high desire to manage MV patient with light sedation, regardless the levels of ventilator setting or the severity of organ dysfunction; answer “C” was defined as low desire to use light sedation for MV patients even if the level of ventilator settings or the severity of organ dysfunction was evidently low; and answer “B” was defined as the desire for using light sedation depending on the clinical condition. Based on suggestion of a panel of experts, answers A, B, and C were scored 1, 0, and −1 to represent physician's desire for light sedation from high to low, respectively. The sum of score for each specific item in the questionnaire was used to evaluate the physician's propensity score for light sedation (PS-_LS_, theoretically ranged from −10 to 10). Thereby, ICU mean physician's PS-_LS_ (ICU-_mean_PS-_LS_, i.e., the sum of physicians' PS-_LS_ divided by the number of the interviewed physicians in the ICU) was used to estimate the overall propensity for light sedation of each participating ICU where the cross-sectional study was successfully completed.

### Design of the Cross-Sectional Study

A 24 h cross-sectional study was conducted in the ICUs participating questionnaire survey on May 11th, 2016. Inclusion criteria were adult MV patients equal to or older than 18 years old and the predicted ICU stay over 24 h. Exclusion criteria included MV patients <18 years old or GCS = 3 and pregnant females. In addition to baseline and demographic data, we collected information regarding the previous MV days, the original disease, Acute Physiology and Chronic Health Evaluation (APACHE II) score and the clinical outcome. Variables related to mechanical ventilation and organ dysfunction listed on the questionnaire were recorded at 6:00 a.m., 14:00 p.m., and 22:00 p.m., respectively while RASS was assessed. RASS ≥ +2 and RASS −2 to 1 were used to define agitation and light sedation. Patients with 1 record of RASS ≤ -3 were categorized into the poorly maintained at light sedation group while those with 3 records of RASS ≥-2 were allocated into well-maintained at light sedation group.

In literature, there are lack of criteria to evaluate nociceptive stimulus. Naturally, intensity of stimulus might change with the variety of stimulus sources, such as the 10 events listed on this questionnaire. Therefore, the practical measurements of all these variables were used to semi-quantitatively assess stimulus intensity of what the recruited patients suffered, named as semi-quantitative stimulus intensity (SSI) score. We predefined SSI as 2, 1, and 0 if the observed value of each specific item was equal to or above the level of answer “A,” “B,” or “C,” respectively.

### Statistical Analysis

All data were double logged and proofread by Epidata 3.1 and a database was developed. Quantitative data were described using mean (SD) or median (IQR) and categorical variables using frequencies and percentages. Normal distribution of data was analyzed by Kolmogorov-Smirnova test or Shapiro-Wilk test. We analyzed relevant covariates that might associate with the probability of being well-maintained at light sedation and mortality in MV patients with univariable and multivariable binary logistic regression and reported odds ratios with 95% CIs and *p*-values. Chi-square test was used for trend of the prevalence of patients well-maintained at light sedation in ICU-_mean_PS-_LS_ quartiles. The relationship between probability of well-maintaining patients at light sedation and the estimated SSI score was analyzed using Pearson correlation. All statistical analyses were performed using SPSS software (V.18.0, Chicago, IIIinois, USA). A *p*-value below 0.05 (2-sided significance testing) was considered statistically significant.

## Results

### Demographic Characters of the Interviewed Physicians and the Recruited Patients

The questionnaire was successfully completed by 558 out of 576 (96.9%) interviewed physicians, comprising of 166 (29.7%) residents, 221 (39.6%) attending, and 171 (30.7%) senior physicians in 102 ICUs among 77 hospitals located in 26 Chinese cities. Demographic data of physicians and characteristics of the ICUs and hospitals are summarized in Table 2. Out of these 102 participating ICUs, however, 10 ICUs were excluded owing to the absence of adult MV patients (*n* = 7), all patients with GCS = 3 (*n* = 1) and two or less physicians of the ICU completing the questionnaire (*n* = 2) in this cross-sectional study. A total of 749 MV patients were included from the remaining 92 ICUs, with age of 62.9 ± 18.2 years old, APACHE II score of 16 (11–22) and 5 (1–16) days receiving MV before the study day (Table 2).

**Table 2:** Demographics of the interviewed physicians and recruited MV patients.

**ICU physicians (*****N*** **=** **558)**		**Recruited patients (*****N*** **=** **749)**	
Male, *n* (%)	311 (55.7)	Male, *n* (%)	512 (68.4)
Years of experience <10, *n* (%)	349 (62.5)	Age, mean (SD), *y*	62.9 ± 18.2
From hospital with beds <2,000, *n* (%)	193 (34.6)	^b^Surgical disease, *n* (%)	402 (53.7)
From general ICU, *n* (%)	347 (62.2)	APACHE II score, median (IQR)	16 (11–22)
From ICU with beds <20, *n* (%)	193 (34.6)	Receiving MV days, median (IQR)	5 (1–16)
^a^From ICU with nurses/bed <2.5:1, *n* (%)	280 (50.2)	Sedation practices for MV patients	
From ICU with MV cases/y <1,000, *n* (%)	414 (74.2)	Well-maintained at light sedation, *n* (%)	459 (61.3)
Professional title		Poorly maintained at light sedation, *n* (%)	290 (38.7)
Resident, *n* (%)	166 (29.7)	Agitation, *n* (%)	226 (27.0)
Attending, *n* (%)	221 (39.6)	Agitation in lightly-sedated cases, *n* (%)	161 (35.1)
Senior, *n* (%)	171 (30.7)	^c^Cases of death, *n* (%)	184 (24.6)

a *Ratio of nurses/bed lower than 2.5:1 meant nurse shortage in these ICUs*.

b *Surgical disease was referred as ICU admission from operating room or surgical departments*.

c *Cases of death: all recruited patients were followed up for 28 days, and 2 cases were lost*.

### Concern of ICU Physician on Light Sedation

**Figure 1:**
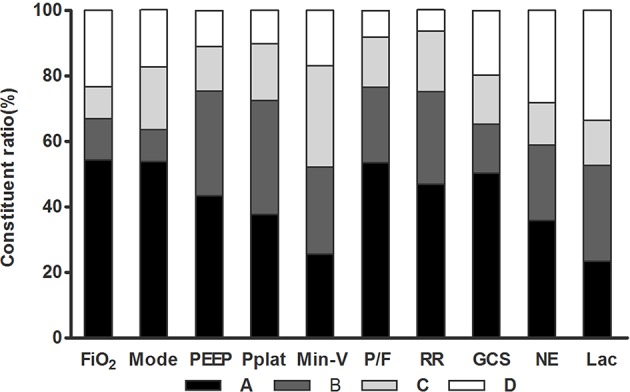
Constituent ratio of physician's options for the 10 items in the questionnaire. FiO_2_, fraction of inspiration O_2_; Mode, ventilation mode; PEEP, positive end-expiratory pressure; Pplat, plateau pressure; Min-V, minute ventilation; P/F, PaO_2_/FiO_2_; RR, respiratory rate; GCS, Glasgow Coma Scale; NE, dosage of norepinephrine or equal-effect dose of other vasopressors; Lac, plasma lactate.

The majority of the recruited ICU physicians were concerned of ventilator setting [520/558 (93.2%)] and disease severity [554/558 (99.3%)] when titrating the sedation depth. Only three physicians (0.5%) answered that neither ventilator settings nor organ dysfunction severity impacted their sedation practices. For each item in this questionnaire, 66.4–93.7% of the physicians chose the answer of A, B, or C. That is, only 6.3–33.6% of them chose answer D (Figure 1).

### ICU Physician's PS-_LS_ and ICU-_mean_PS-_LS_

For each specific item, the physician's response varied greatly (Figure 1). In particular, answer “A” was selected by over 50% of the physicians in four events: “Mode,” “FiO_2_,” “PaO_2_/FiO_2_,” and GCS, respectively. On the other hand, choosing answer “C” varied from 9.8% (FiO_2_) to 31.1% (Min-V).

**Figure 2:**
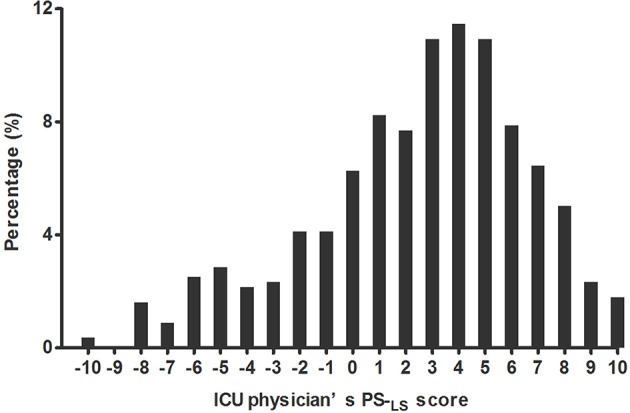
Distribution of ICU physicians at different propensity scores for light sedation (PS-_LS_). The physician's PS-_LS_ ranged from −10 to 10 with median (IQR) 3 (0–5), which were non-normally distributed by Kolmogorov-Smirnova test (*P* < 0.001). The larger PS-_LS_ score was, the more interviewees preferred light sedation.

The median (IQR) of physicians' PS-_LS_ was 3 (0–5) ranging from −10 to 10 (Figure 2). Among the 558 interviewees, only 46 (8.2%) received PS-_LS_ ≤ −5 (i.e., who selected answer C for five specific events at least), while 192 physician (34.4%) had PS-_LS_ ≥5 (i.e., who selected answer A for five specific events at least). Most physicians (320, 57.4%) had no intense propensity for the sedation level. Among all analyzed characters, only the gender (female vs. male, β = 0.713; 95%CI: 0.016–1.411; *p* = 0.045) was significantly associated with PS-_LS_ (Table S2). Interestingly, the physician seniority (residents, attending, or senior physician) did not affect distribution of ICU physicians in different PS-_LS_ (*p* = 1.000, Figure S1).

Based on PS-_LS_ of the interviewed physicians, ICU-_mean_PS-_LS_ was calculated in 92 ICUs participating in cross-sectional study, which was normally distributed (*p* = 0.189), ranged from −5 to 7 with a median (IQR) of 2.37 (0.16–4.33) as shown on Figure S2. Accordingly, the 92 ICUs were divided into quartiles by ICU-_mean_PS-_LS_ (Q1: −5–0.16, 17 ICUs; Q2: 0.16–2.37, 26 ICUs; Q3: 2.37–4.33, 28 ICUs; Q4: 4.33–7, 21 ICUs).

### Risk Factors for Poorly Maintaining MV Patients at Light Sedation

Among the enrolled patients (*n* = 749), 459 (61.3%) patients were well-maintained at light sedation levels (RASS ≥ −2). Meanwhile, 38.7% (290/749) patients were recorded with RASS ≤ -3 at least once within the 24 h observational period (Table 2). Multivariable binary logistic regression demonstrated that risk factors for poorly maintaining MV patients at light sedation included APACHE II score (OR, 1.039; 95% CI: 1.015–1.064; *p* = 0.001), SSI score (OR, 1.502; 95% CI: 1.382–1.633; *p* < 0.001), and ICU-_mean_PS-_LS_ (referred to Q1, Q4 OR, 0.400, 95% CI: 0.239–0.670, *p* < 0.001, Table 3). In addition, the prevalence of well-maintaining MV patients at light sedation was negatively correlated with SSI scores in this cross-sectional study (*r* = −0.967, *p* < 0.001, Figure S3).

**Table 3:** Odds ratios of variables for poorly-maintained light sedation in MV patients (*n* = 749).

	**Total no**.	**Poorly maintained light sedation no. (%)**	**Univariate analysis**		**Multivariate analysis**	
			**OR (95% CI)**	***P*-value**	**Adjusted OR (95% CI)**	***P*-value**
Age in years			0.997 (0.989–1.005)	0.528	0.999 (0.989–1.009)	0.807
Gender						
Male	512	189 (36.9)			Reference	
Female	237	101 (42.6)	1.269 (0.927–1.737)	0.137	1.407 (0.989–2.003)	0.058
Category of diseases						
Surgical diseases	402	161 (40.0)			Reference	
Medical diseases	347	129 (37.2)	0.886 (0.659–1.190)	0.421	0.810 (0.562–1.167)	0.258
^a^Receiving MV days			0.998 (0.996–1.001)	0.167	0.999 (0.996–1.001)	0.296
APACHE II score			1.048 (1.027–1.069)	<0.001	1.039 (1.015–1.064)	0.001
^b^SSI score			1.487 (1.374–1.609)	<0.001	1.502 (1.382–1.633)	<0.001
^c^ICU-_mean_PS-_LS_				0.002		0.001
Q1	142	66 (46.5)	Reference		Reference	
Q2	167	72 (43.1)	0.873 (0.556–1.369)	0.553	1.003 (0.606–1.661)	0.991
Q3	252	100 (39.7)	0.758 (0.500–1.148)	0.190	0.891 (0.558–1.423)	0.629
Q4	188	52 (27.7)	0.440 (0.278–0.697)	<0.001	0.400 (0.239–0.670)	<0.001

*All variables associated with poorly-maintained light sedation in mechanically ventilated patients were illustrated by univariate or multivariable logistic regression model*.

a *Receiving MV days meant days of receiving mechanical ventilation before enrollment*.

b *SSI score, semi-quantitative stimulus intensity score, calculated by ventilator setting and organ dysfunction as predefined in methods*.

c *ICU-_mean_PS-_LS_, ICU mean physician's PS-_LS_ (i.e., the sum of physicians' PS-_LS_ divided by the number of the interviewed physicians in the ICU). The 92 ICUs were divided into quartiles by ICU-meanPS-_LS_ (Q1: −5–0.16; Q2: 0.16–2.37; Q3: 2.37–4.33; Q4: 4.33–7). MV, mechanically ventilated; APACHE, acute physiology and chronic health evaluation*.

### Impact of ICU-_mean_PS-_LS_ on Implementation of Light Sedation in Mechanically Ventilated Patients

**Figure 3:**
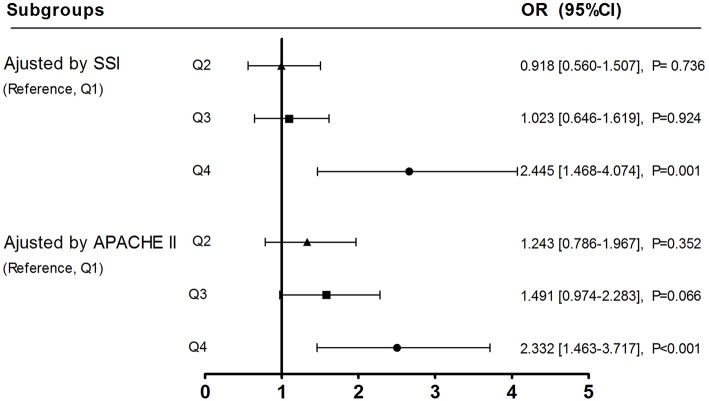
Adjusted odds ratios for ICU_−mean_PS-_LS_ affecting probability of well-maintaining MV patients at light sedation. All the 92 ICUs were divided into quartiles (Q1–4) by ICU_−mean_PS-_LS_ marked as ▴ for Q2, □ for Q3 and • for Q4, respectively. Referred to Q1, OR (95% CI) for probability of well-maintaining MV patients at light sedation was analyzed over increasing ICU-meanPS-LS quartiles (subgroups of Q2, 3, and 4), adjusted by SSI or APACHE II score.

A significant increasing trend in prevalence of MV patients being well-maintained at light sedation was observed over increasing ICU-_mean_PS-_LS_ quartiles (from Q1–Q4, *x*^2^ test for trend, *p* = 0.002). Moreover, odds ratio for probability of being well-maintained at light sedation remained significant in MV patients from Q4 ICUs vs. Q1 ICUs, adjusted by APACHE II score (OR, 2.332; 95% CI: 1.463–3.717; *p* < 0.001) and SSI score (OR, 2.445; 95% CI: 1.468–4.074; *p* = 0.001, Figure 3). In subgroup analysis determined by SSI, furthermore, OR for probability of well-maintained at light sedation in the highest vs. the lowest ICU-_mean_PS-_LS_ quartile was significant in subgroups of low SSI scores (SSI = 0–2, OR, 2.385; 95% CI: 1.025–5.549; *p* = 0.044, Q4 vs. Q1) and middle SSI scores (SSI = 3–5, OR, 2.784; 95% CI: 1.343–5.774; *p* = 0.006, Q4 vs. Q1), but not with high SSI scores (SSI = 6–11, OR, 2.017; 95% CI: 0.649–6.271; *p* = 0.226, Figure S4).

### Risk of Death in MV Patients Poorly vs. Well-Maintained at Light Sedation

**Figure 4:**
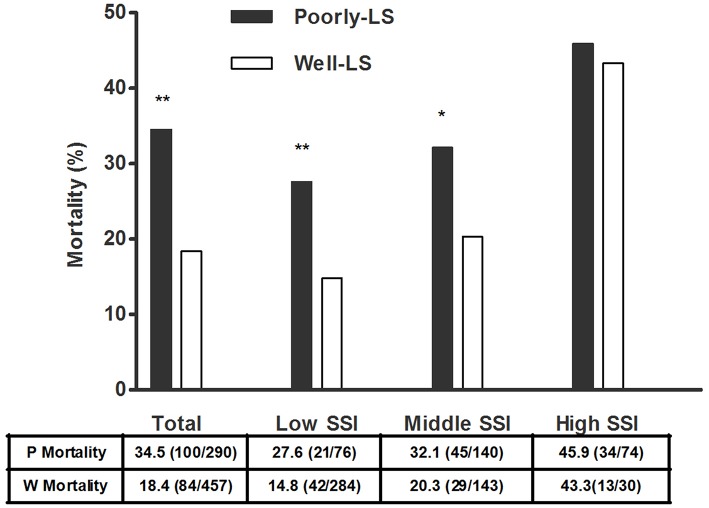
Mortality in MV patients poorly vs. well-maintained at light sedation. Based on the quartiles of SSI scores, 749 MV patients were stratified into low SSI (quartile 1, SSI = 0–2), middle SSI (quartile 2, SSI = 3–5) and high SSI (quartile 3 and 4, SSI = 6–11) subgroups (Patients distributed in quartile 3 and quartile 4 of SSI scores were assigned into high SSI scores subgroup owing to a small number of MV patients in both quartiles). Mortality between MV Patients poorly and well-maintained at light sedation was analyzed by Chi-square test. Poorly-LS meant “poorly-maintained at light sedation” group and Well-LS meant “well-maintained at light sedation” group. ^**^p < 0.01 and ^*^p < 0.05 compared to group Well-LS. SSI, semi-quantitative stimulus intensity; MV, mechanically ventilated.

By a regression model, adjusted OR for mortality was significant in patients characterized with high APACHE II score (OR, 1.072; 95% CI: 1.047–1.097; *p* < 0.001) and receiving deep sedation once at least during 24 h observation period (OR, 2.034; 95% CI: 1.435–2.884; *p* < 0.001). In addition, an association between poorly maintained at light sedation and mortality remained significant in subgroup of MV patients with low SSI (*p* = 0.009) as well as middle SSI (*p* = 0.023), but not in those with high SSI scores (*p* = 0.808, Figure 4).

## Discussion

In this study, we found that almost all of the interviewed physicians were concerned about patients' tolerance levels regarding sedation, while titrating sedation depth for MV patients. Estimated by the responses to all items of the questionnaire, meanwhile, their propensity score for light sedation (PS-_LS_) was highly varied. Importantly, a significant increasing trend in prevalence of MV patients being maintained at light sedation was observed over increasing ICU-_mean_PS-_LS_ quartiles (from Q1 to Q4). Moreover, adjusted odds ratio for well-maintaining MV patients at light sedation remained significant in the highest referred to the lowest ICU-_mean_PS-_LS_ quartile, adjusted by APACHE II score and SSI score. These findings suggested that ICU physician's perception for the tolerance levels regarding sedation of MV patients in light sedation was transferred into clinical practice.

In recent years, the pain, agitation, and delirium guidelines (PAD) (7) and early comfort using analgesia, minimal sedatives and maximal humane care (eCASH) concepts encouraged ICU physicians to maintain the MV patients at lighter sedation levels (21, 22). Timely monitoring RASS and CPOT/BPS (Critical care Pain Observation Tool/Behavior Pain Scale) was recommended to appropriately evaluate patients' tolerance levels regarding sedation toward intensive cares and to effectively avoid excessive sedatives and analgesics (7, 21, 23). Our finding of a shift in distribution of physicians toward preferring light sedation (Figure 2) added additional evidence regarding the impact of guidelines on changes in clinical habits. However, this change remained limited. Prevalence of deep sedation was continually high in MV patients. Similar to other recently published results (10, 11, 24), 38.7% of MV patients were poorly maintained at light sedation in this cross-sectional study (one record of deep sedation at least in 24 h, Table 2). It was noted that the decreased prevalence of deep sedation was predominantly documented in previous RCTs (4–6, 19) rather than in real clinical practices as reported by observational studies (10–12, 25). These data suggested that implementation of the light sedation strategy challenges what intensive care currently delivers.

This study provided the new information that the ICU physicians' concern about patients' tolerance levels in light sedation was an important barrier to implementation of minimizing sedation strategy for MV patients. Generally, titration of analgesics and sedatives was used to regulate patients' tolerance levels regarding sedation against nociceptive stimuli (26). Meanwhile, the actual necessity for the regulation of patients' tolerance levels regarding sedation with analgesics and sedatives remained unclear owing to the lack of criteria to scale stimulus. Light sedation is only contraindicated for distinctive types of patients who are evidenced with extremely high intensity of stimuli, such as patients with serious Acute Respiratory Distress Syndrome (ARDS) or patients presented with severe traumatic brain injury (TBI), etc. (27–29). ICU physicians are not clearly guided to titrate levels of sedation, adapting to various stimuli in the vast majority of MV patients. Faced with frequent agitation in MV patients (Table 2) (18, 19), ICU physicians' concerns about patient tolerance levels regarding sedation against stimuli became inevitable. Moreover, their concerns were highly varied because of individualized estimates of stimuli. Indeed, we found that most physicians (320/558, 57.4%) had no intense propensity for light levels of sedation while 8.2% (46/558) of ICU physicians showed an unjustifiable low desire to use light sedation for MV patients (Figure 2). Therefore, the development of a reliable and valid tool to scale patient's suffering stimuli is necessary for avoiding physicians' concerns about patients' tolerance levels regarding sedation as well as for optimizing the levels of sedation objectively and appropriately. We attempted to scale stimulus what MV patients suffered by a semi-quantitative assessment of the suspected stimulus-derived events, named as semi-quantitative stimuli (SSI) in this cross-sectional study. It was interestingly demonstrated that probability of light sedation was negatively correlated with SSI scores of MV patients (*r* = −0.967, *p* < 0.001, Figure S3). However, further research is needed to promote validity of this tool in stimulus assessment for optimizing our sedation practices.

A strength of this study was the combination of the questionnaire survey with the cross-sectional study, which determined the association between ICU physicians' perception for the tolerance levels regarding sedation of MV patients and his/her decision making for the use of light sedation. In fact, there was previously little evidence regarding whether ICU physicians' beliefs drove sedation practices (30). In this study, a significantly increased prevalence of lightly sedated MV patients was found in ICUs stratified into the highest vs. the lowest ICU-_mean_PS-_LS_ quartile (Table 3). Moreover, this finding was further convinced by adjusting SSI score (Figure 3). These results suggested that ICU physicians' perception of the tolerance levels regarding sedation of MV patients in light sedation impact sedation practice for mechanically ventilated patients was an important factor in the poor implementation of light sedation strategy. Therefore, the development of a valid assessment of nociceptive stimulus to predict rather than to concern patients' tolerance levels regarding sedation against stimuli individually would be helpful to ICU physicians delivering necessity-based sedation depth for MV patients.

Consistent with previous reports, the risk potential of death was significantly increased in deeply sedated patients (1–3). Interestingly, subgroup analysis demonstrated that there was no significant difference in the mortality of MV patients between poorly and well-maintained at light sedation in the high SSI group (SSI ≥ 6). However, poorly maintaining MV patients at light sedation was shown to be harmful for the majority of MV patients, who were estimated with low and middle SSI. Notably, the impact of ICU-_mean_PS-_LS_ on the prevalence of light sedation was significant in these two subgroups of MV patients. ICU physicians' concern of patients' tolerance levels regarding sedation in light sedation seemed to be associated with outcomes.

There were several limitations in this study. Firstly, the 10 specific events of the questionnaire were identically weighted to analyze PS-_LS_. However, the answer “D” (an issue considered unimportant for sedation depth decision-making) could partially, at least, diminish the influence of this limitation. Secondly, the MV patients' suffering stimuli were multiple and complex. Only few variables regarding ventilator settings and vital organ dysfunction were considered in this study. However, those 10 events were derived from a Delphi processing and were approved by the interviewees. Thirdly, we didn't investigate the association of physicians PS-_LS_ with their decision making on depth of sedation for MV patients, for whom they were responsible. Indeed, it was too difficult to be performed reliably. Despite this, this study provided a window to look at the perception of ICU physicians on light-sedated patients' tolerance levels regarding sedation vs. their clinical behaviors.

## Conclusions

Owing to lack of criteria to scale stimulus, ICU physicians' perception for patients' tolerance levels regarding sedation in light sedation was highly individualized. Importantly, the fact that a significantly increasing prevalence of well-maintaining MV patients at light sedation was observed over increasing ICU-_mean_PS-_LS_ quartiles suggested that ICU physicians' perception for the tolerance levels regarding sedation of MV patients in light sedation affected decision making on sedation depth for MV patients. As the consequence of ICU physicians' individualized concerns, poorly maintenance at light sedation was frequent, and was significantly associated with an increased mortality. Therefore, to develop a valid assessment for nociceptive stimuli rather than to individually analyze concerns about patients' tolerance levels regarding sedation would be helpful for ICU physicians delivering necessity-based sedation depth for MV patients. SSI score was an attempt to scale stimulus in this study. Further research is needed to promote its reliability and validity.

## Data Availability Statement

All datasets generated for this study are included in the manuscript/Supplementary Files.

## Ethics Statement

The study was conducted in accordance with the Helsinki Declaration and approved by the Ethics Committees of each participating hospital. Owing to the absence of additional interventions, the requirement for written informed consent was waived by the ethics committees of all participating hospitals. The study protocol was registered on the website of www.chictr.org.cn (registration number: ChiCTR-EOC-16008444).

## Author Contributions

PM contributed to the study concept and design, data interpretation, and article drafting. YG contributed to the literature search, questionnaire drafting, data collection, data analyzation, and article drafting. HY and JL participated in questionnaire drafting, data collection, and information organization. JX participated in the study design and data statistical analyzation. JZ participated in the study design. All authors read and approved the final manuscript.

### Conflict of Interest

The authors declare that the research was conducted in the absence of any commercial or financial relationships that could be construed as a potential conflict of interest.

## Abbreviations

MV: mechanically ventilated
RASS: Richmond Agitation Sedation Scale
RCTs: randomized control trials
Mode: ventilation mode
PEEP: positive end-expiratory pressure
Pplat: plateau pressure
FiO_2_: fraction of inspiration
RR: respiratory rate
Min-V: minute ventilation
P/F: PaO_2_/FiO_2_
Lac: plasma lactate level
GCS: Glasgow Coma Score
NE: norepinephrine
PS-_LS_: propensity score for light sedation
ICU-_mean_PS-_LS_: ICU mean physician's PS-_LS_
APACHE II: Acute Physiology and Chronic Health Evaluation II
SOFA: Sequential Organ Failure Assessment
SSI: semi-quantitative stimuli Intensity
PAD: Pain, Agitation, and Delirium
eCASH: early Comfort using Analgesia, minimal Sedatives and maximal Humane care
CPOT/BPS: Critical care Pain Observation Tool/Behavior Pain Scale
ARDS: Acute Respiratory Distress Syndrome
TBI: Traumatic brain injury.
