# Supplementary material for: ICU Physicians' Perception of Patients' Tolerance Levels in Light Sedation Impacts Sedation Practice for Mechanically Ventilated Patients
Source: Front Med (Lausanne). 2019 Oct 18;6:226. doi: 10.3389/fmed.2019.00226 (PMC6813223; doi:10.3389/fmed.2019.00226)
Supplement: Supplementary file 1 [file Data_Sheet_1.PDF]

Supplemental materials

Table S1 Concerns of ICU physicians on lightening sedation depth for MV patients by the modified Delphi consensus meeting

Items	Consensus of experts by Delphi processing			Test
	First round	Second round	Third round	
Mode	15/15 (100.0%)	14/15 (93.3%)	15/15 (100%)	55/63(87.3%)
PEEP	14/15 (93.3%)	15/15 (100.0%)	15/15 (100%)	56/63(88.9%)
Pplat	13/15 (86.7%)	14/15 (93.3%)	14/15 (93.3%)	52/63(82.5%)
FiO_2	15/15 (100.0%)	14/15 (93.3%)	14/15 (93.3%)	57/63(90.5%)
RR	13/15 (86.7%)	15/15 (100.0%)	14/15 (93.3%)	51/63(81.0%)
Min-V	14/15 (93.3%)	14/15 (93.3%)	13/15 (86.7%)	52/63(82.5%)
PO_2/FiO_2	15/15 (100.0%)	15/15 (100.0%)	15/15 (100.0%)	60/63(95.2%)
Auto-PEEP	10/15 (66.7%)	12/15 (80.0%)	12/15 (80.0%)	48/63(76.2%)
$PaCO_2$	8/15 (53.3%)	10/15 (66.7%)	9/15 (60.0%)	38/63(60.3%)
GCS	14/15 (93.3%)	15/15 (100%)	15/15 (100.0%)	51/63(81.0%)
NE	15/15 (100.0%)	14/15 (93.3%)	14/15 (93.3%)	52/63(82.5%)
Lac	14/15 (93.3%)	11/15 (73.3%)	13/15 (86.7%)	51/63(81.0%)
$ScVO_2$	9/15 (60.0%)	8/15 (53.3%)	9/15 (60.0%)	41/63(65.1%)
Cr	8/15 (53.3%)	7/15 (46.7%)	8/15 (53.3%)	33/63(52.4%)
ALB	7/15 (46.7%)	8/15 (53.3%)	7/15 (46.7%)	28/63(44.4%)
SOFA	11/15 (73.3%)	10/15 (66.7%)	12/15 (80.0%)	48/63(76.2%)
APACHE II	11/15 (73.3%)	12/15 (80.0%)	11/15 (73.3%)	39/63(61.9%)
ISS	7/15 (46.7%)	5/15 (33.3%)	6/15 (40.0%)	26/63(41.3%)

Note: By a follow-up three rounds Delphi processing, ten highly agreed items (with agreement of 13/15 at least) were selected as the major components of this questionnaire. No difference was found in approval of these items between tested doctors and experts ($p>0.05$). Mode: ventilation mode, PEEP: positive end-expiratory pressure, Pplat: plateau pressure, FiO_2: fraction of inspiration O_2, RR: respiratory rate, Min-V: minute ventilation, P/F: PaO_2/FiO_2, Auto-PEEP: automatic positive end-expiratory pressure, $PaCO_2$: partial pressure of carbon dioxide, GCS: Glasgow Coma Score, NE: dosage of norepinephrine or equal-effect dose of other vasopressors, Lac: plasma lactate, $ScVO_2$: systemic central venous oxygen saturation, Cr: creatinine, ALB: albumin, SOFA: Sequential Organ Failure Assessment, APACHE II, Acute Physiology and Chronic Health Evaluation II, ISS: injury severity score

Table S2 Multivariate linear regression analysis for the factors impacting ICU physician's PS-$_{LS}$

Variables	β	95%CI	*P* value
Hospital beds	-0.164	(-0.903,0.575)	0.663
Types of ICU	0.142	(-0.595,0.880)	0.705
ICU beds	0.207	(-0.594,1.008)	0.612
Ratio of nurse/bed	-0.084	(-0.817,0.648)	0.821
ICU-admissions with MV/y	0.164	(-0.665,0.994)	0.698
Gender	0.713	(0.016,1.411)	0.045*
Working years	-0.245	(-1.274,0.785)	0.641
Professional title	0.377	(-0.266,1.020)	0.250

* Compared to males, female interviewees preferred to light sedation.

PS-$_{LS}$: propensity score for light sedation. Hospital beds: ≥2000 vs <2000 beds; Types of ICU: special ICUs vs general ICUs; ICU beds: ≥20 vs <20 beds; Ratio of nurse/bed: ≥2.5:1 vs <2.5:1, ICU-admissions with MV/yr: ≥1000 vs <1000; Working years: ≥10 vs <10. Professional title was classed into senior, attending and resident.

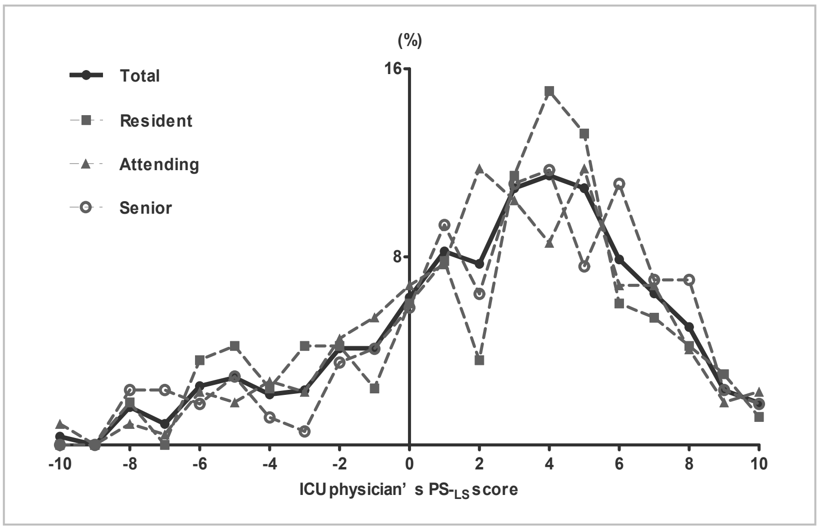

Figure S1. Distribution of physicians' seniority at different propensity scores for light sedation (PS-$_{LS}$)

558 interviewees were allocated into groups of senior physician, attending physician and resident, respectively. Ps-$_{LS}$: propensity score for light sedation. Dotted line with symbol circle: Ps-$_{LS}$ in senior physicians. Dotted line with symbol triangle: Ps-$_{LS}$ in attending physicians. Dotted line with symbol square: Ps-$_{LS}$ in residents. There was no difference between different professional titles of interviewees (p = 1.000).

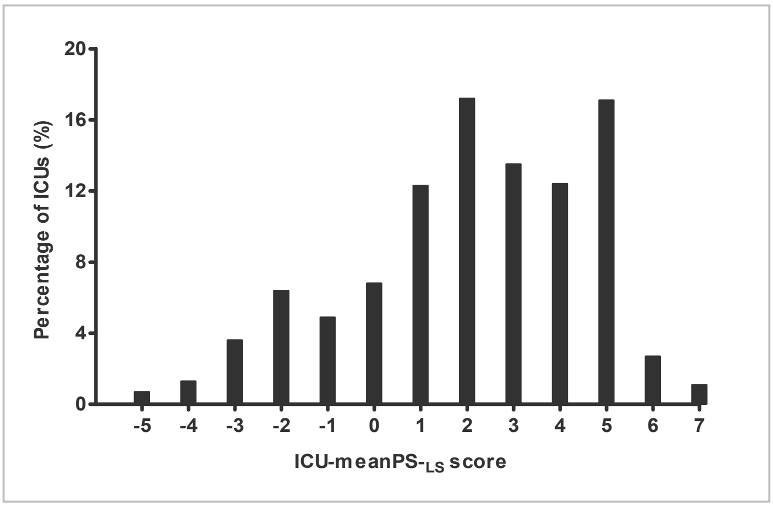

Figure S2. Percentages of ICUs classified with ICU-$_{mean}$PS-$_{LS}$ score

The ICU-$_{mean}$PS-$_{LS}$ score ranged from -5 to 7 (The larger the score, the higher the propensity) with median (IQR) 2.37(0.16-4.33) in the recruited 92 ICUs. The score -5 referred to scores from -5 to < -4 subsection, and so on, for each of the others. ICU-$_{mean}$PS-$_{LS}$: ICU mean physician's PS-$_{LS}$.

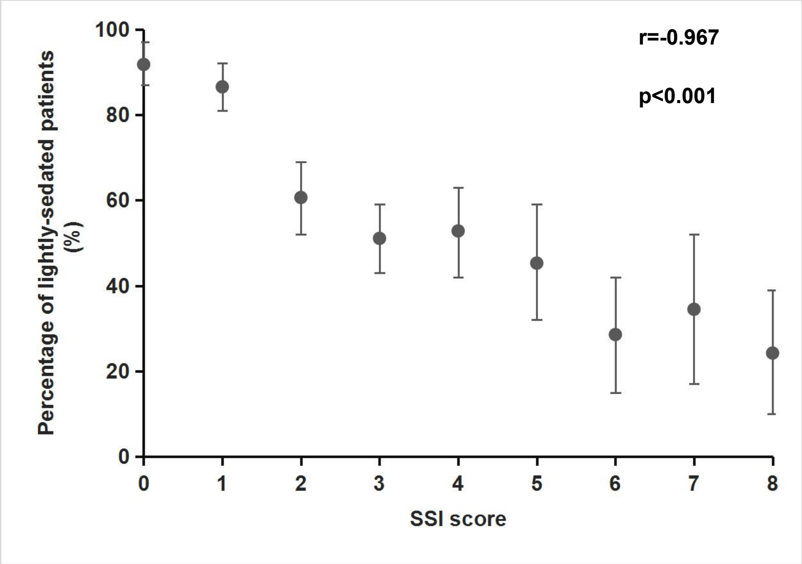

Figure S3 Correlation between the percentage of lightly-sedated patients and the estimated SSI score

The prevalence of patients maintained at light sedation (RASS ≥ -2) with estimated SSI score was presented by percentages (white line)

and 95% confidence interval (CI, black zones). Patients with SSI ≥ 8 were analyzed together due to the small number of patients. SSI: semi-quantitative stimulus intensity.

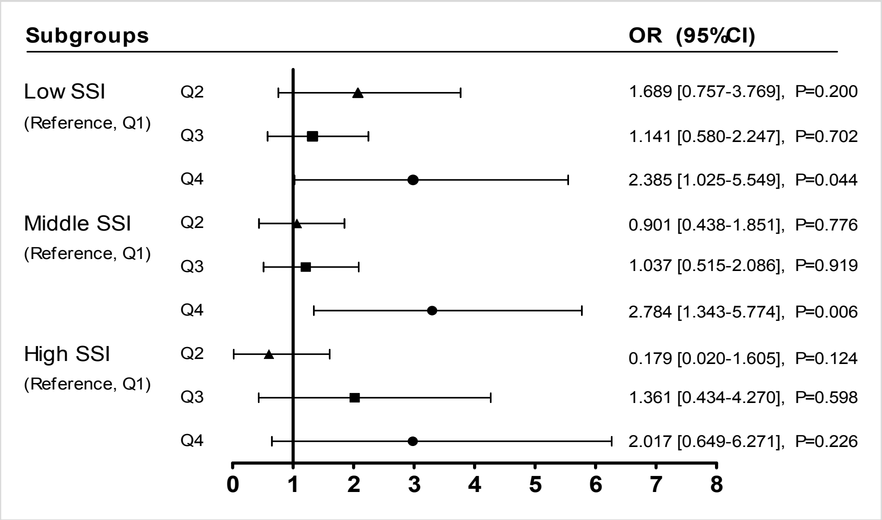

Figure S4 Odds ratios for probability of well-maintained light sedation over ICU$_{-mean}$PS-$_{LS}$ increasing quartiles in SSI subgroups

A total of 92 ICUs were divided into quartiles by ICU-meanPS-$_{LS}$ marked as ▲ for Q2, ■ for Q3 and ● for Q4 respectively. All the recruited 749 mechanically ventilated (MV) patients were stratified into subgroups of low SSI scores (SSI = 0-2, n = 362), middle SSI scores (SSI = 3-5, n = 283) and High SSI scores (SSI = 6-11, n = 104) respectively (Patients distributed in quartile 3 and quartile 4 of SSI scores were assigned into high SSI scores subgroup together owing to a small number of MV patients). Taking Q1 as the reference, the probability of well-maintained light sedation in Q2, 3 and 4 was presented by OR(95% CI).
